# Editorial: Rosaceae Fruit Development and Quality

**DOI:** 10.3389/fpls.2021.837300

**Published:** 2022-01-20

**Authors:** Chunying Kang, Jia-Long Yao, Zhongchi Liu, Yuepeng Han

**Affiliations:** ^1^Key Laboratory of Horticultural Plant Biology (Ministry of Education), Huazhong Agricultural University, Wuhan, China; ^2^Hubei Hongshan Laboratory, Wuhan, China; ^3^The New Zealand Institute for Plant and Food Research Ltd, Auckland, New Zealand; ^4^Department of Cell Biology and Molecular Genetics, University of Maryland, College Park, MD, United States; ^5^Key Laboratory of Plant Germplasm Enhancement and Specialty Agriculture, Wuhan Botanical Garden, The Innovative Academy of Seed Design, Chinese Academy of Sciences, Wuhan, China

**Keywords:** Rosaceae, fruit development, fruit ripening, metabolites, post-harvest, database

Rosaceae is a large angiosperm family consisting of ~3,000 species, which contains many economically important fruit crops, such as apple, pear, peach, strawberry, apricot, and cherry. These crops are grown worldwide and provide humans with diverse foods and essential nutrition. There exist a series of fundamental biological questions to address in fruit crops, for instance, the regulation of ripening, aroma and flavor, etc. With the advances of high throughput sequencing and other genomics technologies, it becomes possible to investigate the basic regulatory mechanisms underlying fruit specific biological processes. This Research Topic showcases exciting findings in Rosaceae fruits ranging from male sterility, fleshy fruit development, fruit ripening, metabolite biosynthesis, to post-harvest quality regulation.

Male sterility is a valuable agronomic trait utilized for hybrid crops. In peach, male sterility also frequently occurs. To better understand the underlying reasons, the cytological and physiological changes during microspore development were carefully investigated in the male sterile variety “Jinxiang,” a popular yellow flesh peach cultivar in China (Cai et al.). The study found that an increase in reactive oxygen species (ROS) levels along with a decrease in antioxidant levels may cause abnormal development of microspores and tapetum, resulting in male sterility in peach.

In flower development, the four whorls of floral organs were specified by the coordination of ABCE class genes. Many studies showed that these floral identity genes also play important roles in fruit development. Yao et al. summarized recent advances on this aspect, with an emphasis on findings in apple, pear and strawberry.

Fruit ripening can be grouped into two types, climacteric and non-climacteric. The former type is mainly controlled by the hormone ethylene and has been extensively studied. However, the mechanism of non-climacteric ripening is not well-known. Fruit ripening in strawberry undergoes a typical non-climacteric ripening process. Bai et al. summarized the central roles of the ABA-controlled ripening mainly using strawberry as a model. The interactions of ABA with ethylene, IAA, polyamines, and sugars were discussed. In addition, this review also discussed the involvement of ABA in the ripening of climacteric fruit. Besides hormones, autophagy also takes part in fruit ripening. Autophagy is a catabolic and recycling pathway that maintains cellular homeostasis under normal growth and stress conditions. Sánchez-Sevilla et al. discovered autophagy-related structures and expression of autophagy-related genes (*ATG*) in the fruit flesh cells of strawberry. Furthermore, fruit ripening was delayed by blocking autophagy either biochemically or genetically.

Fruit size, flavor, and aroma are important characteristics of fruit quality. In apple, a bud sport mutant with larger fruit was previously identified. Bu et al. found that the content of free auxin was increased and expression levels of several auxin pathway genes were altered in the fruit of the bud sport mutant, suggesting that auxin plays an important role in increasing fruit size. Strawberry fruit is rich in volatile compounds. One of these compounds, γ-decalactone (γ-D), has the greatest contribution to the characteristic fruity aroma in strawberry. Previous study revealed that γ-D biosynthesis is controlled by a single gene *FaFAD1*, but the exact mutations occurring in different strawberry varieties remained unknown. Oh et al. uncovered the genomic variations of *FaFAD1*, determined a positive effect of *FaFAD1* allele dosage on the γ-D content by employing the bacterial artificial chromosome (BAC) library in cultivated octoploid strawberry, and developed genetic markers for breeding strawberry cultivars with high volatile contents.

To identify important loci regulating different physiological and fruit quality traits in peach, a multi-locus genome wide association study (GWAS) was performed using 620 individuals (da Silva Linge et al.). Dozens of quantitative trait nucleotides (QTNs) were identified and validated, which would support the development of DNA tools for peach breeding. García-Gómez et al. characterized the fruit quality traits and measured the contents of main metabolites, and then generated and analyzed transcriptomes of fruit flesh at three developmental stages during ripening of two apricot varieties, which differ in fruit color, soluble solid content, and firmness. With the combination of these data, *Carotenoid Cleavage Dioxygenase 4* (CCD4) and *Sucrose Synthase* (SS) were identified as the candidate genes of the light yellow/white fruit color and high soluble solid content during the ripening process.

Optimal postharvest treatments are critical for maintaining fruit quality. Loquat (*Eriobotrya japonica*) fruit flesh accumulates lignins when the fruit suffers chilling injury during postharvest storage. Ge et al. revealed a new regulatory step during this process. They found that the MADS-box protein EjAGL65 could bind to and inhibit the activity of the promoter of *EjMYB8*, encoding a positive regulator of lignin biosynthesis. Under excessive postharvest chilling, expression of *EjAGL65* is reduced and *EjMYB8* becomes active, which leads to accumulation of unwanted lignins.

Availabilities of high-quality reference genomes and transcriptome data are valuable resources for both basic and applied researches. Li et al. summarized the progresses on the latest genome assemblies and annotations of major Rosaceae crop species and provides a list of websites hosting these data. This review is a useful guide to researchers working in the fields of Rosaceae fruits.

## Author Contributions

CK prepared the first draft of this editorial. J-LY, ZL, and YH revised the editorial. All authors approved the editorial for publication.

## Funding

This work was supported by the National Key Research and Development Program of China (2018YFD1000102, 2019YFD1000800), National Natural Science Foundation of China (31822044), and PFR Growing Futures Fund.

## Conflict of Interest

Author J-LY was employed by company the New Zealand Institute for Plant and Food Research Ltd. The remaining authors declare that the research was conducted in the absence of any commercial or financial relationships that could be construed as a potential conflict of interest.

## Publisher's Note

All claims expressed in this article are solely those of the authors and do not necessarily represent those of their affiliated organizations, or those of the publisher, the editors and the reviewers. Any product that may be evaluated in this article, or claim that may be made by its manufacturer, is not guaranteed or endorsed by the publisher.

